# Sequence and gene expression evolution of paralogous genes in willows

**DOI:** 10.1038/srep18662

**Published:** 2015-12-22

**Authors:** Srilakshmy L. Harikrishnan, Pascal Pucholt, Sofia Berlin

**Affiliations:** 1 Swedish University of Agricultural Sciences, Department of Plant Biology, Uppsala BioCenter, Linnean Centre for Plant Biology, P.O. Box 7080, SE-75007 Uppsala, Sweden

## Abstract

Whole genome duplications (WGD) have had strong impacts on species diversification by triggering evolutionary novelties, however, relatively little is known about the balance between gene loss and forces involved in the retention of duplicated genes originating from a WGD. We analyzed putative Salicoid duplicates in willows, originating from the Salicoid WGD, which took place more than 45 Mya. Contigs were constructed by *de novo* assembly of RNA-seq data derived from leaves and roots from two genotypes. Among the 48,508 contigs, 3,778 pairs were, based on fourfold synonymous third-codon transversion rates and syntenic positions, predicted to be Salicoid duplicates. Both copies were in most cases expressed in both tissues and 74% were significantly differentially expressed. Mean Ka/Ks was 0.23, suggesting that the Salicoid duplicates are evolving by purifying selection. Gene Ontology enrichment analyses showed that functions related to DNA- and nucleic acid binding were over-represented among the non-differentially expressed Salicoid duplicates, while functions related to biosynthesis and metabolism were over-represented among the differentially expressed Salicoid duplicates. We propose that the differentially expressed Salicoid duplicates are regulatory neo- and/or subfunctionalized, while the non-differentially expressed are dose sensitive, hence, functionally conserved. Multiple evolutionary processes, thus drive the retention of Salicoid duplicates in willows.

Whole genome duplication (WGD or polyploidy) has long been recognized as an important evolutionary force that create biological novelty and complexity^1^,^2^ and polyploidization is regarded as the trigger for the rapid diversification of angiosperms^3^,^4^,^5^. In fact, most flowering plant lineages have undergone one or more rounds of ancient polyploidization events^4^,^5^. For example, the *Arabidopsis thaliana* genome shows signs of two recent WGDs and one triplication event that is likely shared by all eudicots^6^,^7^,^8^ and the poplar, *Populus trichocarpa* genome shows signs of this ancient triplication event, as well as a more recent WGD^9^. A WGD introduces massive genetic redundancy, as immediately after the WGD, every gene will have a copy, and the two copies are often referred to as paralogs or duplicates. Over evolutionary times, polyploid genomes can undergo diploidization^10^,^11^ (also known as fractionation^12^), making the genomes more diploid-like. Since the duplicates have redundant functions immediately after they are formed, one of the duplicate copies might become nonfunctional by accumulating deleterious mutations (nonfunctionalization or pseudogenization), without any effects on fitness of the individual. During diploidization, therefore, massive gene loss and genome rearrangements usually take place.

Many duplicates, however, escape nonfunctionalization and are retained even long after the WGD, which is for example seen in the cotton (*Gossypium raimondii*)^13^ and poplar (*P. trichocarpa*) genomes^9^ that contain approximately 2,000 and 8,000 pairs of retained paralogous genes respectively. The genes retained in duplicates are assumed to follow distinctive modes of evolution, resulting in functional diversification or conservation of redundant function. According to the neofunctionalization model^1^,^14^, the degenerating copy escapes nonfunctionalization by acquiring a substitution that lead to a new gene function, which is expected to be fixed by drift^15^, meanwhile, the other copy retains the ancestral function. Following neofunctionalization, the functions of the copies are expected to be maintained by purifying selection^15^. The subfunctionalization, or more specifically the duplication-degeneration-complementation (DDC) model^14^, proposes that both copies acquire degenerate substitutions that damage their functions, however both will eventually be fixed in the population by drift^15^. As neither of the copies can perform its original function, that is, they are subfunctionalized, both will be maintained by purifying selection. Both neo- and subfunctionalization are thus expected to lead to functional diversification of the retained duplicates, which can be changes in regulatory regions, leading to differential expression or to changes in the coding sequences, resulting in differences in protein functions.

Subfunctionalization can also be manifested as the partitioning of expression of the duplicates between different developmental stages or between different tissues. Examples of rapid subfunctionalization by tissue-specific reciprocal silencing have previously been demonstrated^16^,^17^,^18^. A third possible outcome for retained duplicates is that their functions are conserved, throughout evolutionary times, meaning that the duplicates escape functional diversification. Some classes of genes, for example transcription factors and genes involved in signal transduction are overrepresented among duplicates with conserved redundant functions^19^. It is hypothesised that these genes are sensitive to changes in expression levels and therefore must be present in the same number of genomic copies as the genes with whose product they interact (summarized in the gene balance model^20^,^21^).

The Salicoid WGD is the most recent WGD that has been detected in poplars (Genus: *Populus*)^9^,^22^. Interestingly, this WGD is shared with the willows (genus: *Salix*)^9^,^23^, suggesting that it should have taken place before the divergence of the two lineages, more than 45 million years ago (Mya)^24^,^25^. As sequence divergence was estimated to be around three times higher between willow and poplar duplicates (with a predicted origin from the Salicoid WGD) compared to orthologs^23^, it suggests that the WGD happened long before the speciation event (assuming a constant molecular clock). In a more recent study based on whole-genome sequence data, the divergence time was estimated to 52 Mya and the WGD to six million years prior to the lineage split^26^. These estimates, therefore suggest that the Salicoid WGD is relatively ancient and happened sometime between 45 and 58 Mya. Both willow and poplar genomes are organized in nineteen chromosomes (n = 19) and they display high levels of macrosynteny, although several major rearrangements have been detected^23^. Willow genomes are generally smaller than poplar genomes and they also contain fewer protein coding genes. For example, the predicted genome size of *P. trichocarpa* is 485 million base pairs (Mbp) and the genome contains 41,335 protein coding genes^9^, while the predicted genome size of *Salix purpurea* is 450 Mbp and contains 37,865 putative protein coding genes (*Salix purpurea* v1.0, DOE-JGI, http://phytozome.jgi.doe.gov/pz/portal.html#!info?alias=Org_Spurpurea). The recently sequenced *Salix suchowensis* genome is possibly even smaller as it was estimated to be 425 Mbp with only 25,599 putative protein coding genes^26^. Based solely on these observations, we therefore expect fewer Salicoid duplicates in willows than in poplars. In order to study mechanisms operating on retained paralogous genes after a WGD, we first aimed to identify retained Salicoid duplicates among expressed genes in willows and secondly to investigate sequence and gene expression divergence between the Salicoid duplicates. This gave us important information on evolutionary processes involved in the retention of duplicated genes after a WGD.

## Results

### The construction and filtering of the *de novo* transcriptome assembly

**Figure 1:**
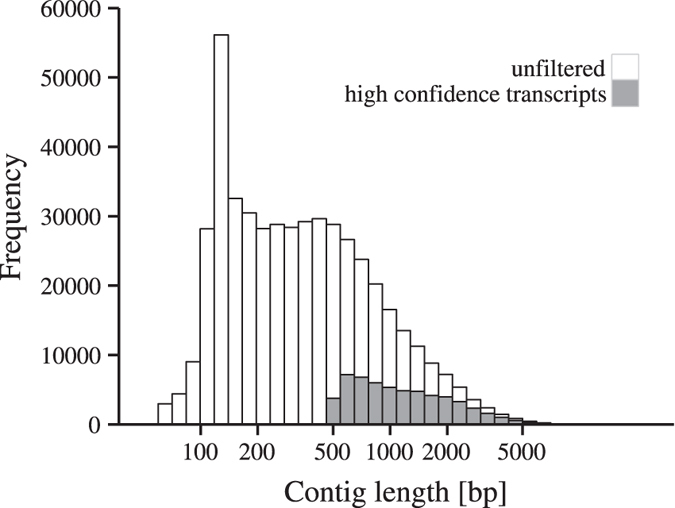
Length distribution of the contigs generated by the Trans-ABySS assembler. The white distribution shows the contig lengths in the unfiltered assembly and the light grey distribution shows the contig lengths in the filtered assembly.

As we generated a total of 769 × 10^6^ 100 bp reads from all the samples, the total output of the reads was 76.9 × 10^9 ^bp, representing about 180-fold of the predicted willow genome size (Table 1). After trimming adapter sequences and removing low-quality bases, 682 million sequencing reads from all samples were combined and a *de novo* assembly was built with Trans-ABySS^27^,^28^. The total number of generated contigs was 392,355 with lengths ranging from 61 to 15,602 bp (Fig. 1). To remove lowly expressed and erroneous contigs, the assembly was filtered on expression levels (at least one fragment per kilobase of contig per million mapped fragments (FPKM)) and on contig lengths (at least 500 bp in length). This resulted in 52,215 significantly expressed contigs, with a minimum length of 500 bp (Fig. 1). These were finally translated to peptide sequences and only contigs with open reading frames were retained, resulting in 48,508 contigs in the final assembly. This filtered assembly was used for further analysis. When the contigs were mapped to the putative protein coding transcripts in the draft genome of *S. purpurea*, 32,563 matches were retrieved. As the *S. purpurea* draft genome contains 37,865 protein coding transcripts, the contigs, thus represent as many as 86% of all protein coding transcripts present in the current version of the *S. purpurea* genome. As a quality control of the assembly, the proportion of sequencing reads that were integrated in the assembly was estimated. The trimmed sequencing reads were mapped back to the unfiltered assembly with 392,355 contigs, resulting in successful mapping of 80.1% of the sequencing reads, of which 73.8% aligned uniquely. This demonstrates that the reads were well represented in the assembly. On the other hand, when the sequencing reads were mapped to the filtered assembly with 48,508 contigs, on average 26.2% of all the trimmed reads mapped (Table 1). Of these, on average 73.2% mapped to unique positions and 58.9% aligned with no mismatches (Table 1). This shows that a large proportion of sequencing reads in the unfiltered assembly were present in short and/or lowly expressed contigs and/or contigs that had no detectable open reading frames, which were thus not present in the filtered assembly.

### Identification and verification of Salicoid duplicates

**Figure 2:**
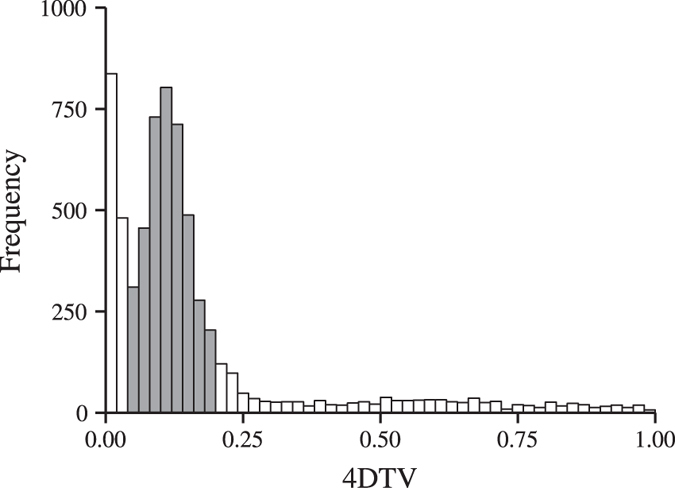
4DTV rates between all predicted duplicates. The predicted Salicoid duplicates are highlighted in light grey.

Putative duplicate pairs were identified by reciprocal BLAST using BLASTP, assuming that for every contig, the second best hit was its duplicate copy (the best BLAST hit was assumed to be to itself). This analysis resulted in 11,279 predicted duplicate pairs. To identify Salicoid duplicate pairs, we estimated sequence divergence, or more specifically, we determined the fourfold synonymous third-codon transversion rate (4DTV) between each pair. Previous studies in *P. trichocarpa*^9^ and *S. suchowensis*^26^ demonstrated that Salicoid duplicate pairs have 4DTV values approximately between 0.1 and 0.2. Guided by the distribution of 4DTV values in Fig. 1, we identified 3,981 pairs in the peak, with 4DTV values between 0.04 and 0.2 (Fig. 2, Supplementary Table S1 online). 203 pairs that were located within 100 kb of each other (using the *S. purpurea* genome as reference) were filtered away, as they, possibly, were tandem duplicates. 3,778 duplicate pairs were therefore predicted to be Salicoid duplicates, which means that 23% of all expressed genes in the leaves and the roots of these willows are retained Salicoid duplicates.

**Figure 3:**
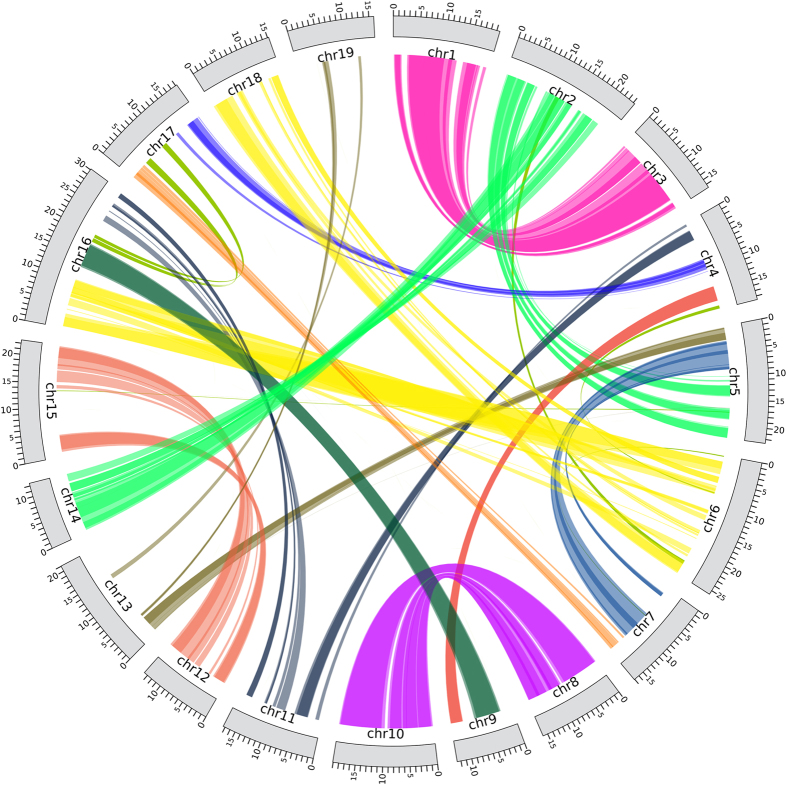
Positions of Salicoid duplicate copies in the *S. purpurea* genome. The lines connect the two copies in every pair. Most Salicoid duplicates are located on homeologous chromosomes originating from the Salicoid WGD, for example on chromosome 8 and 10, 12 and 15, 5 and 7 and on 2, 5 and 14. Homeologous chromosomes were defined in the *P. trichocarpa* genome^9^. The image was created with Circos^46^.

Homeologous genomic segments originating from the Salicoid WGD have been identified in the *P. trichocarpa* genome^9^. For example, several chromosomes are more or less completely homeologous, e.g. chromosome 8 and 10, 12 and 15, while others contain large homeologous segments, e.g. 5 and 7, and 2, 5 and 14^9^. It is thus expected that Salicoid duplicate copies in a pair map to homeologous chromosomes. In order to test this, we mapped each copy to the *S. purpurea* genome, assuming conserved synteny between the willow species as well as between willows and poplars. A total of 6,004 duplicate copies (3,002 pairs) mapped to one of the nineteen chromosomes, while the rest either did not map or mapped to scaffolds. For 2,789 pairs (93%), the two copies were located on different chromosomes and in most cases they were located on chromosomes previously described as homeologous^9^ (Fig. 3). This observation, based on synteny, thus strongly supports that the predicted duplicates indeed are Salicoid duplicates.

### Gene expression divergence of Salicoid duplicates

**Figure 4:**
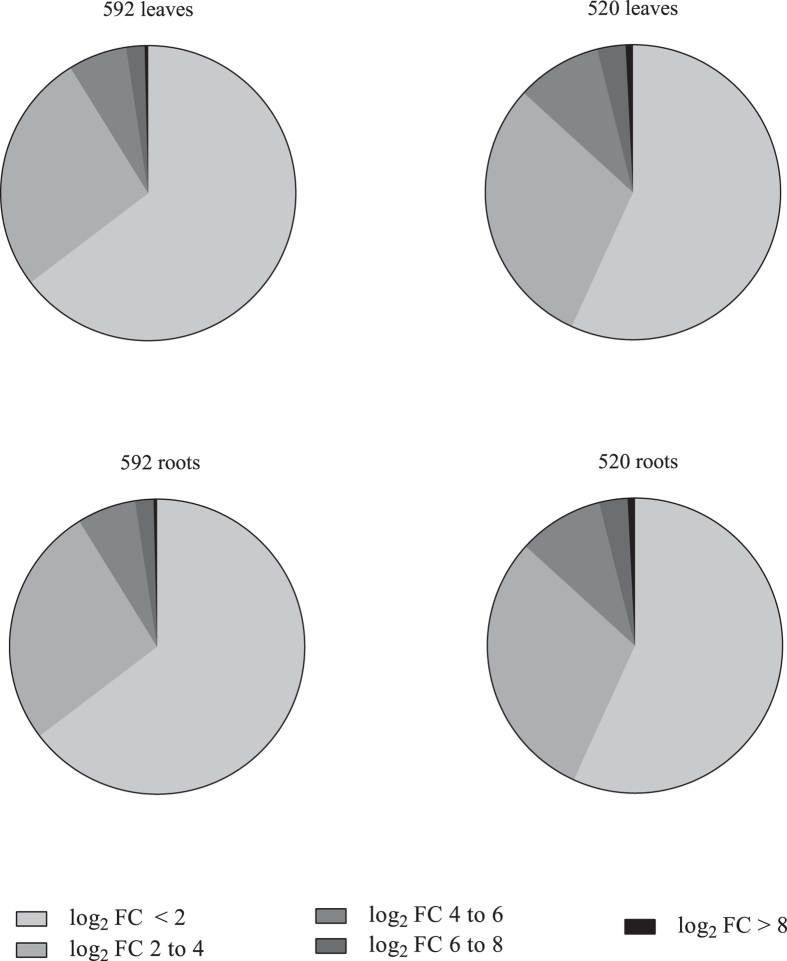
Levels of differential expression between Salicoid duplicates in the two genotypes and tissues. FC = fold change.

To study expression differences i.e. gene expression divergence between the Salicoid duplicate pairs, gene expression was quantified for every Salicoid duplicate copy in the two tissues and genotypes separately (Supplementary Table S2 online). For 3,704 pairs, both copies were expressed in at least one of the tissues and genotypes and for 70 pairs, both copies were expressed in only the leaves and 127 in only the roots, which means that for most Salicoid duplicate pairs both copies were expressed in both tissues. For three pairs, the two copies were expressed in different tissues, thus their expression were partitioned among the tissues. Differential expression between the Salicoid duplicate copies was investigated in the two tissues and genotypes separately (Supplementary Table S3 online). On average, 2,872 (76%) pairs displayed significant levels of differential expression (False discovery rate (FDR) ≤ 0.05) across the tissues and genotypes (520 leaves: 3,079, 520 roots: 2,784, 592 leaves: 3,029 and 592 roots: 2,595). Most copies displayed low levels of differentiation (log_2_ fold change (FC) between 0.4 and 2). Some copies were, however highly differentially expressed (Fig. 4A–D).

### Sequence divergence of Salicoid duplicates

**Figure 5:**
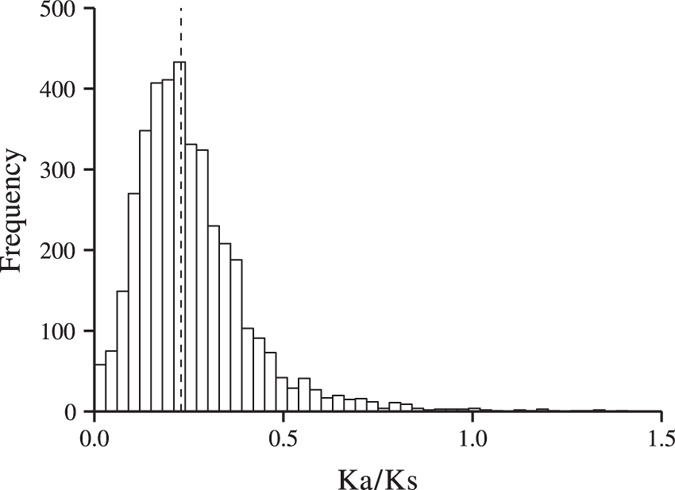
Distribution of Ka/Ks values between the Salicoid duplicates. The median is indicated by the dashed line.

We examined the level of sequence divergence by estimating the Ka/Ks ratio (non-synonymous substitutions per non-synonymous sites/synonymous substitutions per synonymous sites) for every Salicoid duplicate pair. The ratios centred around a peak at 0.23 (Fig. 5), and only 21 of the pairs had Ka/Ks ratios > 1. This indicates overall slow rates of protein evolution at these genes, suggesting that the retained Salicoid duplicates are evolving under purifying selection. To investigate the association between coding-sequence divergence (i.e. Ka/Ks, Ka, Ks and 4DTV) and gene expression divergence we used Spearman Rank correlations. We found a weak, albeit significant positive correlation between Ka/Ks and differential expression, while all other comparisons were non-significant (Table 2). To further investigate the association between coding-sequence and gene expression divergence, we also compared Ka/Ks ratios between different classes of Salicoid duplicates after classification based on level of differential expression and found that Ka/Ks was lowest for the pairs with log_2_ FC < 2 and highest for the Salicoid duplicates with log_2_ FC > 8 (Supplementary Fig. S1 online). These differences were however not statistically significant.

### Functional annotation and GO enrichment analyses

**Figure 6:**
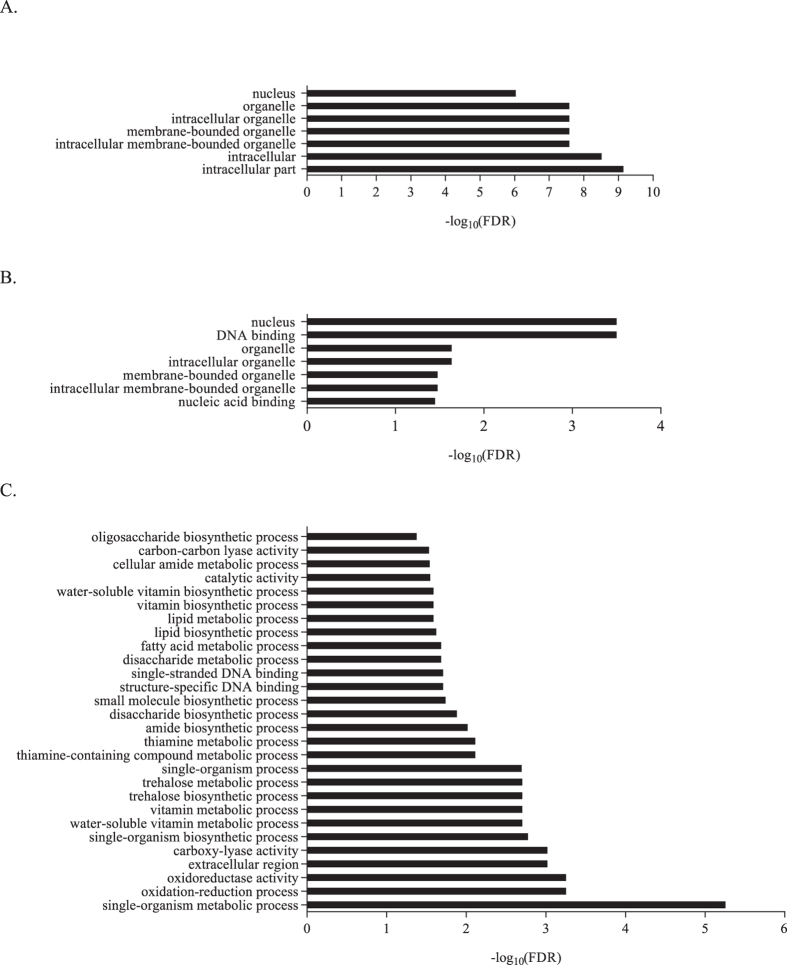
Enriched GO terms. (**A**) All Salicoid duplicates vs. all contigs. (**B**) The non-differentially expressed Salicoid duplicates vs. all contigs. (**C**) The differentially expressed Salicoid duplicates vs. all contigs.

To functionally classify the contigs, we used the Blast2GO software for annotation and 33% of all contigs and 88% of the Salicoid duplicates were annotated with gene ontology (GO) terms (Supplementary Table S4 online). We found that the two copies in a pair were always annotated with the same terms, which was expected given the high degree of sequence similarities. To investigate if any functional categories were overrepresented among the Salicoid duplicates, we performed GO enrichment analyses using all contigs as reference. First we tested for enrichment in all Salicoid duplicates and found that several GO terms related to intracellular and nucleus components in the cellular component category were overrepresented (Fig. 6A). We then tested for enrichment among the non-differentially expressed Salicoid duplicates and found overrepresentation of for example DNA- and nuclear binding terms (Fig. 6B). Lastly, we tested for enrichment among the differentially expressed Salicoid duplicates and found overrepresentation of several terms related to metabolism and biosynthesis (Fig. 6C).

## Discussion

In this study we present results from analyses of retained duplicates in willows that originate from a relatively ancient genome duplication that occurred more than 45 Mya, prior to the divergence of poplars and willows. For the analyses, we used 48,508 contigs that were generated by *de novo* assembly of RNA-seq data derived from leaf and root tissues of two willow genotypes. The assembly was produced by assembling trimmed sequencing reads using the Trans-ABySS assembler and by filtering on gene expression levels, contig lengths and the presence of open reading frames. The filtered contigs mapped to 32,563 of the 37,865 predicted protein coding transcripts in the *S. purpurea* genome, thus representing as much as 86% of the transcribed part of the *S. purpurea* genome, showing that extensive gene expression is taking place in these willow leaf and root tissues. Similarly, in leaves of *P. trichocarpa,* as many as 33,044 genes were expressed^29^. This demonstrates that RNA-seq data from a few tissues gives a good representation of the transcribed part of willow and poplar genomes.

Among the contigs, 11,279 putative duplicate pairs were identified, of which 3,778 pairs were predicted to be Salicoid duplicates. Our strategy to use RNA-seq data from two tissues to obtain expressed genes and then relying on sequence divergence for the identification of the Salicoid duplicates, is conservative and we therefore do not expect to have sampled all Salicoid duplicates present in the genomes of these willows. The observed number is however surprisingly low (given that we have sampled approximately 86% of all protein coding genes (based on the *S. purpurea* genome)), compared to the nearly 8,000 pairs that were detected in the *P. trichocarpa* genome^9^. It is hence likely that willow genomes contain fewer retained Salicoid duplicates than poplar genomes, which could simply be because they overall have smaller genomes. It is however also possible that willows have lost relatively more Salicoid duplicate copies than poplars, which was suggested by Dai *et al.*^26^. An interesting hypothesis is that the Salicoid WGD actually led to the split of the two lineages, which follows the hypothesis that if gene losses occur independently in different populations this can lead to population differentiation^30^. According to this hypothesis, different duplicate copies might have been lost in the willow and poplar lineages respectively, leading to the retention of different Salicoid duplicate pairs.

For most Salicoid duplicates, both copies were expressed in both tissues, indicating that subfunctionalization was not manifested by the partitioning of expression between the two tissues. When testing for differential expression between the copies, we found that on average 76% of the Salicoid duplicates were significantly differentially expressed and more than 40% were highly differentially expressed (log_2_ FC > 2). Expression divergence manifested as significant differential expression was therefore found for a large fraction of the retained Salicoid duplicates. The high prevalence of differentially expressed Salicoid duplicates in our dataset as well as in poplar^31^, suggests that expression divergence is a key process in retention of Salicoid duplicates across the two lineages. If we hypothesise that the observed expression differences between the copies are functionally meaningful, implying that the differentially expressed Salicoid duplicates are functionally diverged. Following that argument, it is possible that the differentially expressed Salicoid duplicates are neofunctionalized^1^ and/or subfunctionalized, following the predictions of the DDC model^14^. Unfortunately, we cannot distinguish between the two processes of neo- and subfunctionalization, as we do not know the ancestral gene function, before the Salicoid WGD. Interestingly, expression divergence as a result of subfunctionalization and/or neofunctionalization has previously been demonstrated in a diverse range of plant species, for example in cotton (*Gossypium raimondii*)^13^, soybean (*Glycine max*)^32^, maize (*Zea mays*)^33^ and Arabidopsis^34^, thus manifesting the importance of these process in the maintenance of retained duplicates in plants.

In contrast, a substantial fraction of the Salicoid duplicates were not differentially expressed. One possibility is that these duplicates did not have time to become neo- and/or subfunctionalized, however, as expression divergence is expected to evolve rapidly^13^, there should have been enough time for neo- and/or subfunctionalization to have evolved since the Salicoid WGD. A more likely explanation is therefore that the expression of these duplicates is evolutionary conserved, following the predictions of the gene balance model^20^. This is further supported by the observation that the functional categories nucleic acid binding and DNA binding are overrepresented among the non-differentially expressed Salicoid duplicates, which include transcription factors and other regulatory elements that are expected to be dose sensitive^20^. In addition, our analyses of Ka/Ks ratios indicate extensive purifying selection on the non-differentially expressed Salicoid duplicates (Ka/Ks = 0.23) as well as on the differentially expressed (Ka/Ks = 0.24) Salicoid duplicates, which is expected^15^. Similar low values were obtained for Salicoid duplicates in poplars^31^.

We found no strong correlations between differential expression and any of the measures of coding-sequence divergence (Ks, Ka/Ks, Ks och 4DTV) for the Salicoid duplicates, however we observed a weak, but significant, positive correlation between DE and Ka/Ks. Given the low correlation coefficients, it is doubtful that these correlations have any biological meaning, therefore our results, favours the uncoupling of the mechanisms of gene expression and sequence divergence in the Salicoid duplicates. This indicates that substitutions in coding sequences have little impact on expression divergence, suggesting that regulatory substitutions play a more significant role. Interestingly, this fits well with the hypothesis that the observed expression differences are the result of neo- and/or subfunctionalization of regulatory regions.

To conclude, in this study, we have identified retained duplicates with a presumed origin from the Salicoid WGD. We estimated the expression and coding sequence divergence between the Salicoid duplicates and found two classes of duplicates; the differentially expressed and the non-differentially expressed. We hypothesise that the differentially expressed Salicoid duplicates are regulatory neo- and/or subfunctionalized, while the non-differentially expressed are dose sensitive and therefore functionally conserved. This shows that similar evolutionary processes are operating on retained Salicoid duplicates in both willows and poplars^31^. Our analyses, furthermore, suggest that willows have much fewer retained Salicoid duplicates than the poplars. This indicates that neo- and/or subfunctionalization occurred in the period between the WGD and the divergence of the two lineages. However, gene loss following the Salicoid duplication continued independently in two lineages. Whether or not this process played a role in the diversification of the two lineages needs to be investigated further.

## Methods

### Sample collection, RNA extractions and sequencing

Fifteen biological replicates for each of two willow genotypes (520 and 592) were cultivated in a walk-in growth chamber with 20 °C constant temperature, 70% relative humidity and 1 h photoperiod (300 μmol photosynthetically active radiation (PAR) m^−2^ s^−1^) with regular watering. The two genotypes are siblings from a cross between *S. viminalis* x (*S. viminalis* x *S. schwerinii*)^23^. After 69 days, two fully developed young leaves and about one centimetre of several root tips were sampled from each plant and immediately snap frozen in liquid nitrogen and stored at −80 °C while awaiting RNA extractions. Approximately 100 mg of leaves and 30 mg of root tips were used for the RNA extractions, which were performed using a Spectrum Plant Total RNA Kit from Sigma-Aldrich with a On-Column DNase I digestion set, also from Sigma-Aldrich. Eighteen samples were selected for sequencing, representing: i) five biological replicates of 520 leaves, ii) five biological replicates of 592 leaves, iii) four biological replicates of 520 roots, and iv) four biological replicates of 592 roots. The RNA samples were first treated with DNase, after which one library per sample was prepared using Illumina’s TruSeq RNA Sample Prep Kit v1, where polyA-fragments were selected, followed by cDNA synthesis and ligation of amplification and sequencing adapters. Sequencing libraries were individually barcoded and then pooled with nine libraries per lane and sequenced on an Illumina HiSeq 2000 instrument. All samples were sequenced as paired-end with 100 bp read length. Library preparations and sequencing were performed by the SNP&SEQ Technology Platform in Uppsala, Sweden.

### Sequencing read processing and *de novo* assembly

The raw sequencing reads were first pre-processed by trimming the adapters and low quality bases with the software Trimmomatics (Version 0.32)^35^. An average quality of 20 was maintained across the sliding window of four bases (SLIDINGWINDOW:4:20) and a minimum length of 75 bp of the reads after trimming (MINLEN:75) was required. The trimmed sequencing reads from all eighteen samples (i.e. libraries) were combined and assembled *de novo* using the paired end read assembler Trans-ABySS^27^,^28^ with k-mer size of 61, k-cov of 5and otherwise with default settings. The assembly was filtered based on transcript lengths ( ≥500 bp) and gene expression levels, which was achieved by mapping the trimmed reads for all samples to the contigs using the program RNA-Seq by Expectation-Maximization (RSEM)^36^ with default settings. A contig was considered to be expressed if it contained at least one mapped fragment per kilobase of contig per million fragments mapped in total (FPKM). Coding and peptide sequences were generated using the TransDecoder tool from the Trinity package^37^. To investigate the proportion of sequencing reads that were integrated in the unfiltered and the filtered assembly, the sequencing reads from each of the different samples were separately mapped using Bowtie2^38^ with a maximum of two mismatches. Different quality metrics measurements i.e. i) total number of reads, ii) total mapped reads, iii) uniquely mapped reads and iv) reads that mapped to more than one position were calculated.

### Identification of Salicoid duplicates

In order to identify duplicates among the filtered contigs, reciprocal BLAST was performed using BLASTP. Two genes were predicted to be duplicates if the reciprocal second best BLAST hit had an e-value lower than 1 × 10^−10^. The best BLAST hit was always excluded as it was assumed to be to itself. Fourfold synonymous third-codon transversion rates (4DTV) were estimated between the pairs and the peak with 4DTV values between 0.04 and 0.2 were predicted to contain the Salicoid duplicates^9^. The reason for using 4DTV rates and not the total number of synonymous substitutions is that transversions occur much more seldom than transitions, therefore back mutations are less likely to have happened at these positions. To estimate 4DTV rates, the peptide sequences of the predicted duplicates were aligned using the ClustalW algorithm^39^ (Version 2.1, gapopen penalty: 10, gapextension penalty: 1) and the alignments were converted to a codon alignment using PAL2NAL^40^. 4-fold degenerate sites were located using an in-house python script and the ratio of transversion per 4-fold degenerate site was calculated. This raw value was corrected for multiple substitutions according to Hellsten *et al.* (2007)^41^. The genomic positions of the Salicoid duplicates in the *S. purpurea* genome were determined by BLAST and the Salicoid duplicates that occurred within 100 kb of each other were considered to be tandem duplicates and were filtered out.

### Ka/Ks calculations

For each Salicoid duplicate pair, the number of non-synonymous substitutions per non-synonymous sites (Ka), the number of synonymous substitutions per synonymous sites (Ks) and the Ka/Ks ratio were calculated in the codeml program in the PAML package using runmode -2 (Version 4.7a)^42^,^43^.

### Gene expression divergence and its correlation with sequence divergence

Gene expression was quantified by mapping the trimmed reads against every Salicoid duplicate copy using Bowtie^38^ allowing a maximum of two mismatches and applying a seed length of 25 bp. Normalised gene expression measured as transcripts per million (TPM) and expected read counts were calculated separately for each tissue and genotype in RSEM^36^ with default settings. A copy was considered expressed in the tissue and genotype if the average TPM was above 1. We used two different measures of expression divergence. First, we examined the partitioning of gene expression between the duplicates across the two tissues, by counting pairs where both copies were expressed (TPM > 1) in at least one of the tissues and pairs where one copy was expressed in one tissue and the other was expressed in the other tissue. Secondly, in every tissue and genotype, differential expression was tested for between the Salicoid duplicates using the *R/Bioconductor* package *edgeR*^44^ with the expected read counts calculated in RSEM^36^ as input. Duplicates were defined as differentially expressed when the false discovery rate (FDR) was ≤ 0.05. In addition, for all predicted Salicoid duplicates, correlations between differential expression and various estimates of sequence divergence (Ka/Ks, Ka, Ks and 4DTV) were analysed by Spearman rank correlations.

### Gene annotations

The protein primary transcripts and Gene Ontology (GO) annotation terms for the Arabidopsis genome were downloaded from The Arabidopsis Information Resource (TAIR) database v. 10 (https://www.arabidopsis.org). A BLAST search was performed with the filtered assembly against the primary transcripts. The best hits were retrieved based on e-values lower than 1 × 10^−10^ and bitscore above 40. The locus identifiers and their respective gene IDs were extracted from the best hit results. An in-house python script was used to assign GO terms to the willow transcripts based on the BLAST results.

### GO enrichment analysis

GO enrichment analyses were performed for different sets using the Blast2GO software^45^. Blast2GO uses Fischer’s exact test to represent relationships between reference and test data sets. The GO terms with corrected p-values ≤ 0.05 were considered to be overrepresented. The set of all annotated contigs was used as the reference set and analyses were done with three different test sets: i) all annotated Salicoid duplicates, ii) all annotated differentially expressed Salicoid duplicates, and iii) all annotated non-differentially expressed Salicoid duplicates.

## Additional Information

**Data availability**: The raw sequencing reads are available in the European Nucleotide Archive (ENA; www.ebi. ac.uk/ena) with the reference number PRJEB10883.

**How to cite this article**: Harikrishnan, S. L. *et al.* Sequence and gene expression evolution of paralogous genes in willows. *Sci. Rep.* **5**, 18662; doi: 10.1038/srep18662 (2015).

## Supplementary Material

Supplementary Information

Supplementary Table S1

Supplementary Table S2

Supplementary Table S3

Supplementary Table S4

## Figures and Tables

**Table 1:** Summary of Illumina sequencing, assembly and mapping.

	520 leaves	%	520 roots	%	592 leaves	%	592 roots	%	Sum	%
Total no of reads	207,824,532		168,557,233		187,381,473		205,123,179		768,886,417	
Total no of bases (Gbp)	20.8		16.9		18,7		20.5		76.9	
No of reads after trimming	174,156,958		148,667,480		168,291,008		190,426,984		681,542,430	
Average read length after trimming	98.4		98.5		97.9		98.7			
Total mapped reads^a^	64,492,170	37.0^b^	35,511,470	23.9^b^	38,630,740	23.0^b^	39,889,518	20.9^b^	178,523,898	26.2^b^
Total unmapped reads^c^	109,664,788	63.0^b^	113,156,010	76.1^b^	129,660,268	77.0^b^	150,537,466	79.1^b^	503,018,532	73.8^b^
Multi-position matches^e^	19,530,798	30.3^d^	8,648,426	24.4^d^	9,284,534	24.0^d^	10,379,248	26.1^d^	47,843,006	26.8^d^
Unique matches^f^	44,961,372	69.7^d^	26,863,044	75.6^d^	29,346,206	76.0^d^	29,510,270	74.0^d^	130,680,892	73.2^d^

^a^Number of filtered sequencing reads that were aligned to the contigs in the filtered assembly.

^b^Relative to the number of reads after trimming.

^c^Sequencing reads that could not be aligned to the contigs in the filtered assembly.

^d^Relative to the number of mapped reads.

^e^Sequencing reads that aligned to two or more positions in the contigs in the filtered assembly.

^f^Sequencing reads aligned to only one position in the contigs in the filtered assembly.

**Table 2:** Spearman rank correlation coefficients (r) and the level of significance (*P*-value) between differential expression and Ka/Ks, Ka, Ks and 4DTV.

	Ka/Ks	Ka	Ks	4DTV
520 leaves	0.051. *P* < 0.01	0.008, ns.	0.001, ns.	0.013, ns.
520 roots	0.089. *P* < 0.0001	−0.009, ns.	0.0014, ns.	−0.001, ns.
592 leaves	0.039. *P* < 0.05	0.013, ns.	0.016, ns.	0.015, ns.
592 roots	0.087. *P* < 0.0001	−0.013, ns.	−0.001, ns.	−0.003, ns.

ns. = not significant.
