# Supplementary material for: Sequence and gene expression evolution of paralogous genes in willows
Source: Sci Rep. 2015 Dec 22;5:18662. doi: 10.1038/srep18662 (PMC4687058; doi:10.1038/srep18662)
Supplement: Supplementary Information [file srep18662-s1.pdf]

SUPPLEMENTARY INFORMATION FOR

Sequence and gene expression evolution of paralogous genes in willows

Srilakshmy L. Harikrishnan[1†], Pascal Pucholt[1†] & Sofia Berlin[1]*

[†]These authors contributed equally to this work.

[1] Swedish University of Agricultural Sciences, Department of Plant Biology, Uppsala BioCenter, Linnean Centre for Plant Biology, P.O. Box 7080, SE-75007 Uppsala, Sweden.

Correspondence should be addressed to S.B. (sofia.berlin@slu.se)

Supplementary Figure

Figure S1. Ka/Ks ratios of Salicoid duplicates classified based on level of differential expression.

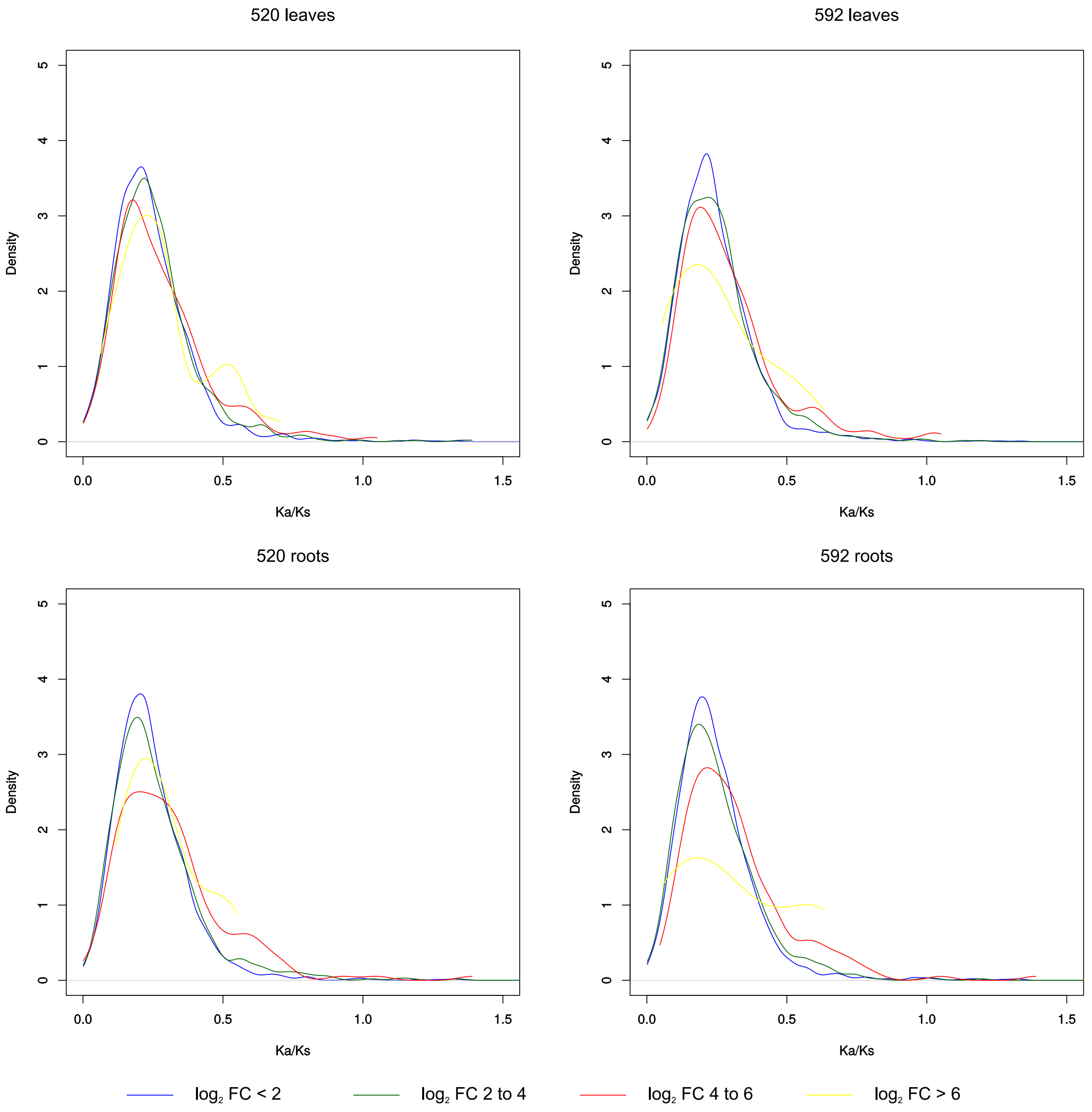

Supplementary Tables

Table S1. All paralogs with Ka, Ks, Ka/Ks and 4DTV values.

(included in a separate excel file)

Table S2. Expression values of Salicoid duplicates for each sample separately.

(included in a separate excel file)

Table S3. Analysis of differential expression between Salicoid duplicates for each sample separately.

(included in a separate excel file)

Table S4. Annotation of contigs in the filtered assembly.

(included in a separate excel file)
